# An ultrasound-induced wireless power supply based on AlN piezoelectric micromachined ultrasonic transducers

**DOI:** 10.1038/s41598-022-19693-5

**Published:** 2022-09-28

**Authors:** Zhicong Rong, Menglun Zhang, Yuan Ning, Wei Pang

**Affiliations:** State Key Laboratory of Precision Measurement Technology and Instruments, Tianjin University, Tianjin, 300072 China

**Keywords:** Energy harvesting, Devices for energy harvesting, Electrical and electronic engineering

## Abstract

Wireless power transfer is one of the enabling technologies for powering implantable biomedical devices. Biocompatibility and CMOS compatibility of wireless power transfer devices are highly desired due to safety and footprint concerns. Toward implantable applications, this paper presents an ultrasound-induced wireless power supply based on AlN piezoelectric micromachined ultrasonic transducer (PMUT). The wireless power supply integrates wireless power transfer, power management and energy storage functions. The PMUT array is used as a passive wireless power receiver, followed by electrical impedance matching networks and a voltage multiplier for efficient power transmission and rectification. The output power intensity of the wireless receiver reaches 7.36 μW/mm^2^ with an incident ultrasound power below the FDA safety limit. The output power of the wireless power supply reaches 18.8 μW and a 100-μF capacitor is fully charged to 3.19 V after power management, which are sufficient to power many low-power implantable biomedical devices such as for neural electrical stimulation, biosensors and intrabody communication applications. The wireless power supply is implemented in a PCB with a diameter of 1 cm. With biocompatibility and CMOS compatibility of AlN thin film compared to commonly used PZT, the proposed solution paves the way for safer and ultraminiaturized wireless power supplies with further development incorporating all the functions on a monolithic chip in the future.

## Introduction

With recent advances in biomedicine, nanotechnology and microelectronics, the demand for wireless power supplies of implantable biomedical devices (IBDs) is rapidly increasing^1^. IBDs are widely applied in daily life, such as neuromuscular stimulators, visual prostheses, cardiac pacemakers, cardiac defibrillators, cochlear implants, pH monitors, blood pressure monitors, and gastrostimulators. These devices can provide real-time diagnosis, treatment and monitoring functions and improve the quality of patients’ life. At present, most implantable biomedical devices still rely on batteries to operate in the human body. Although battery technology has achieved inspiring advancements in recent years^2,3^, this technology still suffers obvious drawbacks. Batteries experience a limited lifetime, relatively high weight and volume, possibility of toxic substance leakage and integration difficulty. Frequent battery replacement for IBD maintenance during treatment could cause inconvenience and possible injury to patients.

To solve these problems, research has been performed to remove batteries from IBDs or prolong the battery lifetime. Wireless power transfer (WPT) is one of the enabling technologies for powering IBDs. Several WPT strategies have been proposed for powering IBDs, mainly including the inductive coupling method, acoustic method, and electromagnetic radiation method^4^. Electromagnetic radiation WPT employs transmitting and receiving antennas to transfer energy through electromagnetic waves^5^. However, electromagnetic waves can easily generate excessive tissue heating, and these waves are highly attenuated in human tissue. Furthermore, the long wavelength of electromagnetic waves results in a relatively large receiver size. Inductive coupling WPT relies on two coupled coils^6^. This method attains a high efficiency in the near field but experiences sharp efficiency deterioration in the far field, limiting the IBD usable depth. Acoustic WPT usually adopts ultrasonic transducers as power receivers. Compared with the other two methods, it can achieve smaller receivers and deeper penetration due to the shorter wavelengths and lower attenuation in the body, respectively^4^. In addition, minimal tissue heating and electromagnetic interference are involved^7^.

In recent years, acoustic WPT researches have attracted much attention^8–13^. Most of these works are devoted to optimizing the power transmission process, or combining the transducer with in vivo applications. Although IBD-orientated acoustic WPT has gained tremendous improvement in recent years, issues and challenges remain^4,6,14,15^. First, most of the piezoelectric receivers currently implemented are based on lead zirconate titanate (PZT), which is not a biocompatible material and potential lead leakage can be harmful to human body and environment. With the increasing awareness of environmental protection and implementation of the Restriction of the Hazardous Substances (RoHS) directive, the use of lead-free and eco-friendly materials to replace lead-containing materials has become a strong trend. Second, a wireless power supply (WPS) integrating WPT with power management and energy storage circuit is usually required for practical applications; however, current PZT ceramic transducers are not IC compatible for a single chip. A single chip integrating CMOS-compatible receiver and CMOS electronics could push a WPS to extreme miniaturization. Compared with these works, the AlN PMUT array adopted in this paper is based on lead-free material and has certain advantages. The fabrication process of AlN thin film is compatible with standard CMOS technology, allowing monolithic integration of MEMS transducers and circuitry^16^. It can be deposited via a low-temperature sputtering process on metalized CMOS wafers. Compared to PZT, the lead-free AlN is a biocompatible material^17–22^. However, current research on AlN PMUT mainly focuses on ultrasonic ranging and ultrasonic imaging applications instead of WPT. Although previous works based on AlN have highlighted possible solutions^23,24^, a complete WPS has not been realized.

Since AlN PMUT-based implantable WPS has not been implemented, this work explores the feasibility of this idea. The proposed WPS integrates WPT and power management functions, including an AlN-based ultrasonic receiver, a voltage multiplier, electrical impedance matching networks, a power management unit, and a charging capacitor. The established WPS is implemented in a 1-cm diameter PCB, and its output power is already sufficient for neural electrical stimulation application in our following step. Since the proposed strategy enables full integration as a monolithic chip in the future, it opens up new ways for wireless and battery-free neural stimulator nodes with substantially reduced footprint as well as improved safety.

## Design and implementation

**Figure 1:**
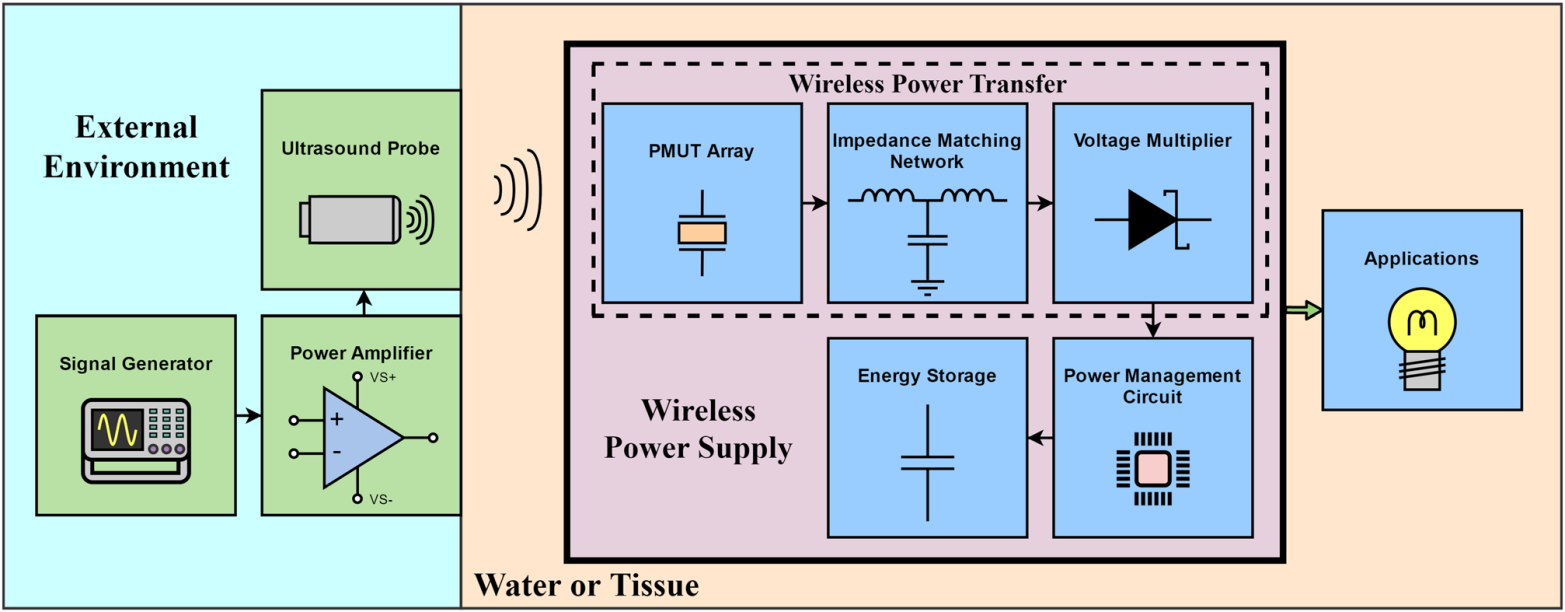
Block diagram of the proposed ultrasound-induced wireless power supply.

The block diagram of the proposed ultrasound-induced wireless power supply (WPS) device is shown in Fig. 1. A function generator is directly terminated with a power amplifier and generates a sinusoidal burst wave to a commercial piezoelectric ultrasonic probe in the external environment. An AlN-based piezoelectric micromachined ultrasonic transducer (AlN-PMUT) array with parylene sealing is designed and fabricated as the wireless power receiver. A 3-stage Villard voltage multiplier is designed and optimized to transform AC into DC power and boost the output voltage with a high efficiency. The power management unit (PMU) is implemented to store and regulate the output DC power of the voltage multiplier. A multilayer ceramic capacitor (MLCC) is employed as the energy storage device. Several passive filter-structured electrical impedance matching networks are designed to improve the power transmission efficiency between the PMUT and subsequent circuit. The whole WPS device is implemented on a low-cost lead-free FR-4 printed circuit board (PCB) substrate.

### PMUT array design, fabrication and characterization

The proposed PMUT element exhibits a two-dimensional stacked multilayer structure consisting of two metal electrodes and a single piezoelectric thin film. Both electrodes comprise molybdenum (Mo), and the piezoelectric layer is an AlN thin film. The proposed AlN-PMUT array is fabricated on a silicon (Si) substrate. When the AlN-PMUT is excited by an incident ultrasonic wave, the piezoelectric thin film vibrates in the flexural mode and a bending moment is generated. The generated bending moment can cause mechanical stress in the AlN piezoelectric film, and then it is converted into an electrical charge through the direct piezoelectric effect.

To obtain higher power transmission efficiency and a relatively smaller volume of the whole device, the resonance frequency of the AlN-PMUT must be properly chosen. First, since the attenuation of sound waves in biological tissue is proportional to the operating frequency, ultrasonic waves with high frequencies attenuate faster and the transmitted energy may not meet the application requirements. Second, when the MEMS transducer is at the Rayleigh distance of the ultrasonic probe, the receiving efficiency is the highest, and the Rayleigh distance of ultrasonic transducers with different frequencies is different, and the frequency can be selected according to the application of different distances. Third, the resonance frequency of the piezoelectric MEMS acoustic wave transducer decreases with the increase of the area and therefore the size of the device also needs to be considered. Because the above three factors need to be considered at the same time, it is important to balance each other according to specific applications. The theoretical resonance frequency of the first mode of the AlN-PMUT can be calculated as^25^:

1 $$\begin{array}{*{20}c} {f = \sqrt {\left( {\frac{3.2}{a}} \right)^{4} \frac{D}{\rho }} /2\pi } \\ \end{array}$$

where *D* is the flexural rigidity of the plate, *a* is the radius of the circular membrane, and *ρ* is the area mass density. According to the above equation, the desired center frequency can be obtained by controlling the thickness of each layer and diameter of the membrane.

**Figure 2:**
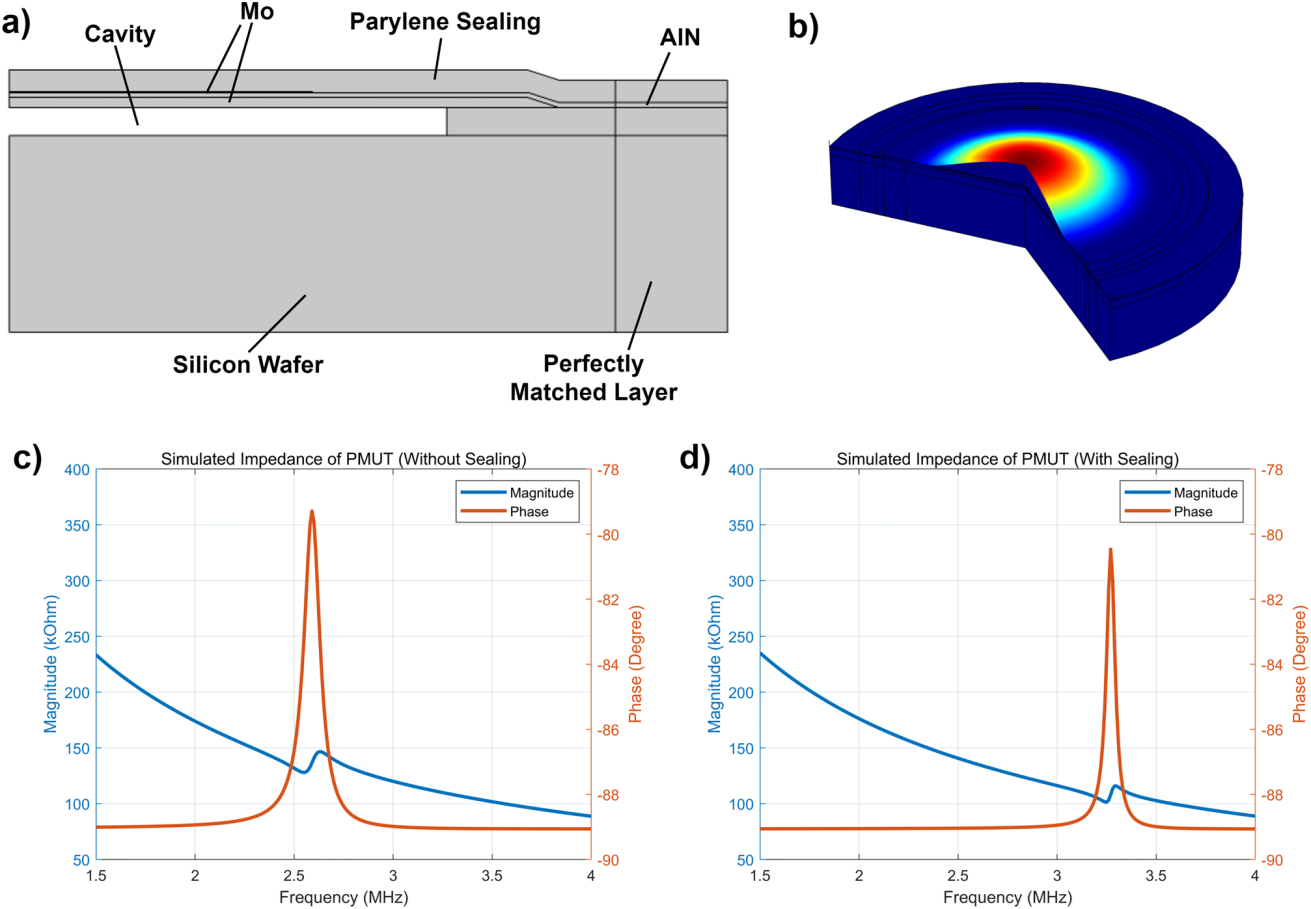
FEA simulation of the AlN-PMUT. (**a**) Two-dimensional FEA simulation model of PMUT. (**b**) Total displacement of the membrane. (**c**) Electrical impedance curve without parylene sealing. (**d**) Electrical impedance curve with 2.2 μm parylene sealing.

For advanced PMUT design, finite element analysis (FEA) was conducted, and the simulation model is shown in Fig. 2a. Due to the symmetry of circular PMUTs, a two-dimensional model was adopted, reducing the computational resource and increasing the computational accuracy. The total displacement of the membrane is shown in Fig. 2b. When the PMUT is used as a receiver, its receiving sensitivity reaches the maximum value when the top electrode coverage is around 70%. According to the simulation results, the receiving performance of the receiver is optimal when the thickness ratio of the piezoelectric layer to the bottom electrode layer is 1:2. In the fabrication, the thickness of the piezoelectric thin film was 0.45 μm, and the thickness of the bottom electrode layer was 0.9 μm. Simulated results of electrical impedance curves are shown in the Fig. 2c,d, with two cases considered, i.e. with and without parylene sealing. According to the simulation results, the phase change at resonance after sealing slightly decreases, and the resonant frequency increases significantly. As the thickness of the parylene sealing increases further, the resonant frequency increases accordingly. The impedance of a single PMUT element is about 10 kΩ, which is very high for a wireless power receiver. To achieve a lower impedance and hence a higher current from the incident ultrasonic waves, many PMUT elements are connected in parallel to form an array. Parallel connection reduces overall impedance from thousands to hundreds of ohms. The resultant PMUT array contains 20 × 20 elements.

**Figure 3:**
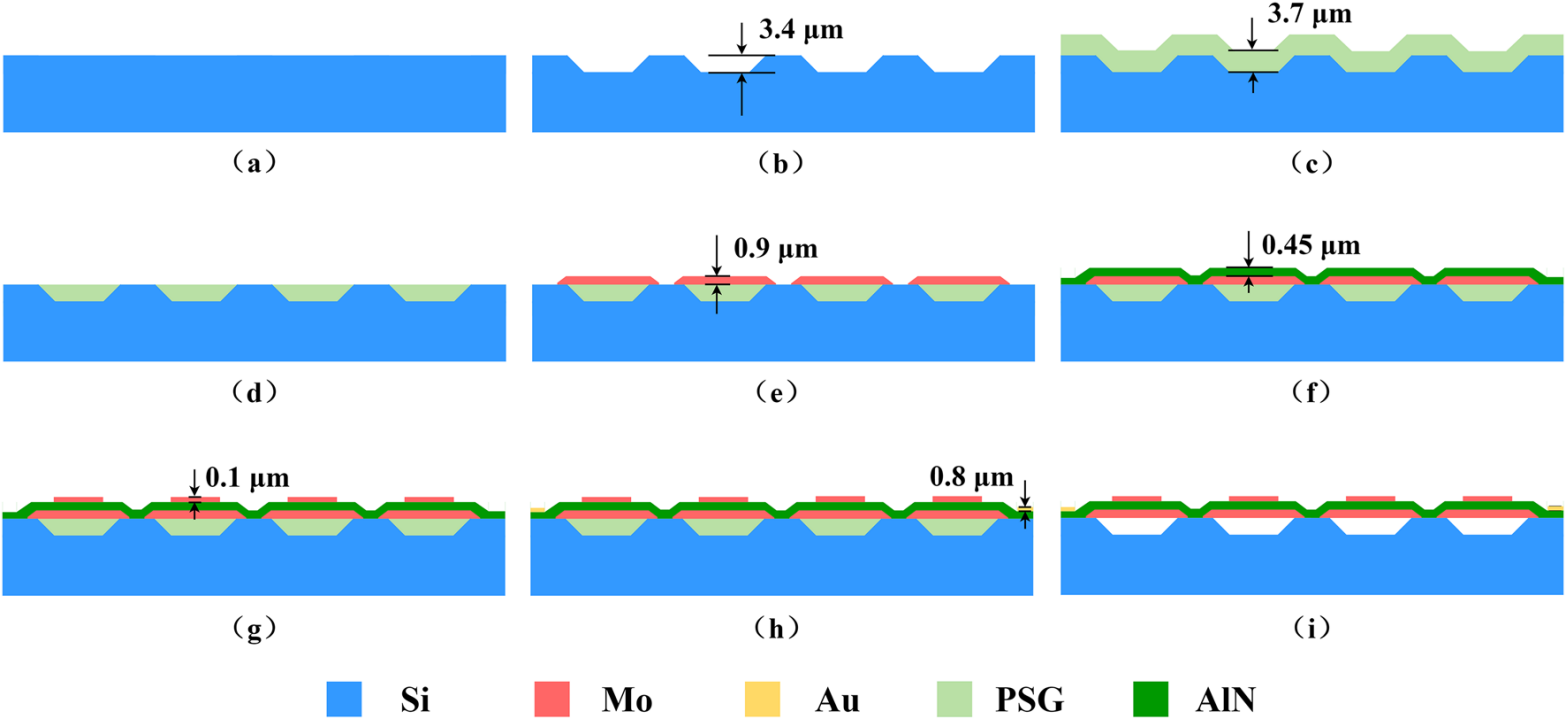
Fabrication process of the proposed AlN-PMUT array. (**a**) Silicon wafer. (**b**) Cavity etching. (**c**) Cavity filling with PSG. (**d**) Planarization of the silicon wafer. (**e**) Deposition and etching of the bottom electrodes. (**f**) Deposition of the AlN thin film. (**g**) Deposition and etching of the top electrodes. (**h**) Deposition of the gold layer as pads. (**i**) PSG etching and structure release.

The fabrication process of the proposed AlN-PMUT array is shown in Fig. 3. First, a high-resistance silicon wafer was used as the substrate and 3.4 μm cavities were etched by reactive ion etching (RIE) process, as shown in Fig. 3a,b. The edges of the cavity were sloped by tuning photoresist profile and RIE etching recipe for better PSG filling in the cavity. PSG with a thickness higher than the cavity depth was deposited by plasma enhanced chemical vapor deposition (PECVD) process, as shown in Fig. 3c. Next, the entire wafer was planarized by chemical mechanical polishing (CMP) process, as shown in Fig. 3d. CMP removed the PSG outside the cavity so that the substrate surface was completely exposed but the PSG in the cavity was left. The uniformity of CMP was less than 10%, which affects the cavity depth. However, the cavity depth has little influence on PMUT performance. Physical vapor deposition (PVD) process was used to deposit 0.9 μm molybdenum as the bottom electrode, as shown in Fig. 3e. The bottom electrode was also patterned into the target shape using the RIE process. Considering that the piezoelectric layer and the top electrode need to be deposited above the bottom electrode, the patterned edge of the bottom electrode was usually a slope, so that the subsequently deposited materials were free from stress concentration. The edges of the bottom electrodes were sloped by tuning photoresist profile and RIE etching recipe. In the next step, PVD process was used to deposit 0.45 μm AlN thin film and 0.1 μm molybdenum top electrode, as shown in Fig. 3f,g. The top electrode and AlN were later patterned. The gold layer was deposited by PVD process with a thickness of 0.8 μm to form connection pads, as shown in Fig. 3h. 0.8 μm gold layer ensures that the step of the etched AlN was fully covered and that the top electrode was connected to the bus. After wafer dicing, the entire wafer was immersed in a hydrofluoric acid solution to remove the PSG and suspend the structure, as shown in Fig. 3i.

**Figure 4:**
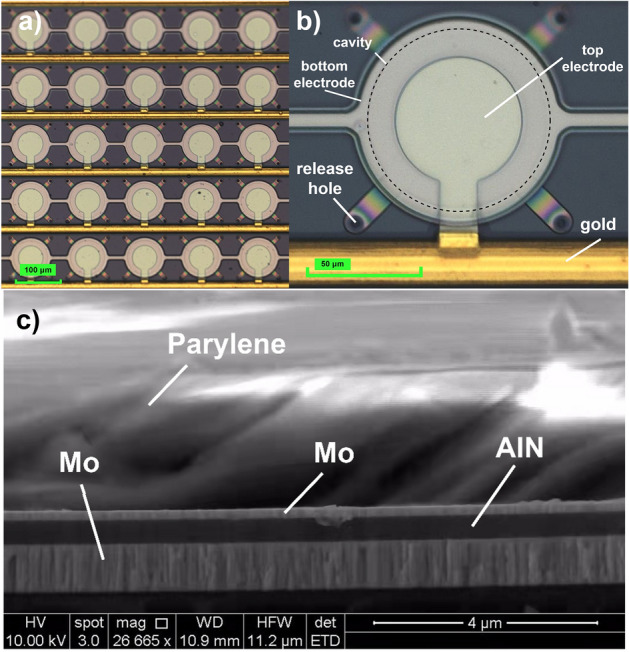
Images of the AlN-PMUT array. (**a**) Microscopic image of the PMUT array. (**b**) Enlarged microscopic image of a PMUT element. (**c**) SEM image of the cross section of the sealed AlN-PMUT.

Figure 4a,b shows optical images of the fabricated AlN-PMUT array. Each individual AlN-PMUT element contains four release holes with a diameter of 10 μm. The diameter of the top electrode was 27 μm and the diameter of the cavity was 39 μm. The distance between each PMUT element was 89 μm. The effective area of the AlN-PMUT array was about 2.55 mm^2^.

Following the unsealed AlN-PMUT array fabrication, a receiving experiment was conducted in a tank filled with deionized water. The output voltage of the PMUT array showed a time-dependent drop, eventually to as low as 100 mV, because the water entered the cavity through the release hole. To solve this issue, the AlN-PMUT array was sealed with parylene. Figure 4c shows an SEM image of the sealed AlN-PMUT array. The thickness of the parylene sealing layer was about 2.1 μm. The cavity was in low vacuum after sealing, and the PMUT receiving sensitivity should increase as the vacuum level further increases due to higher quality factor.

**Figure 5:**
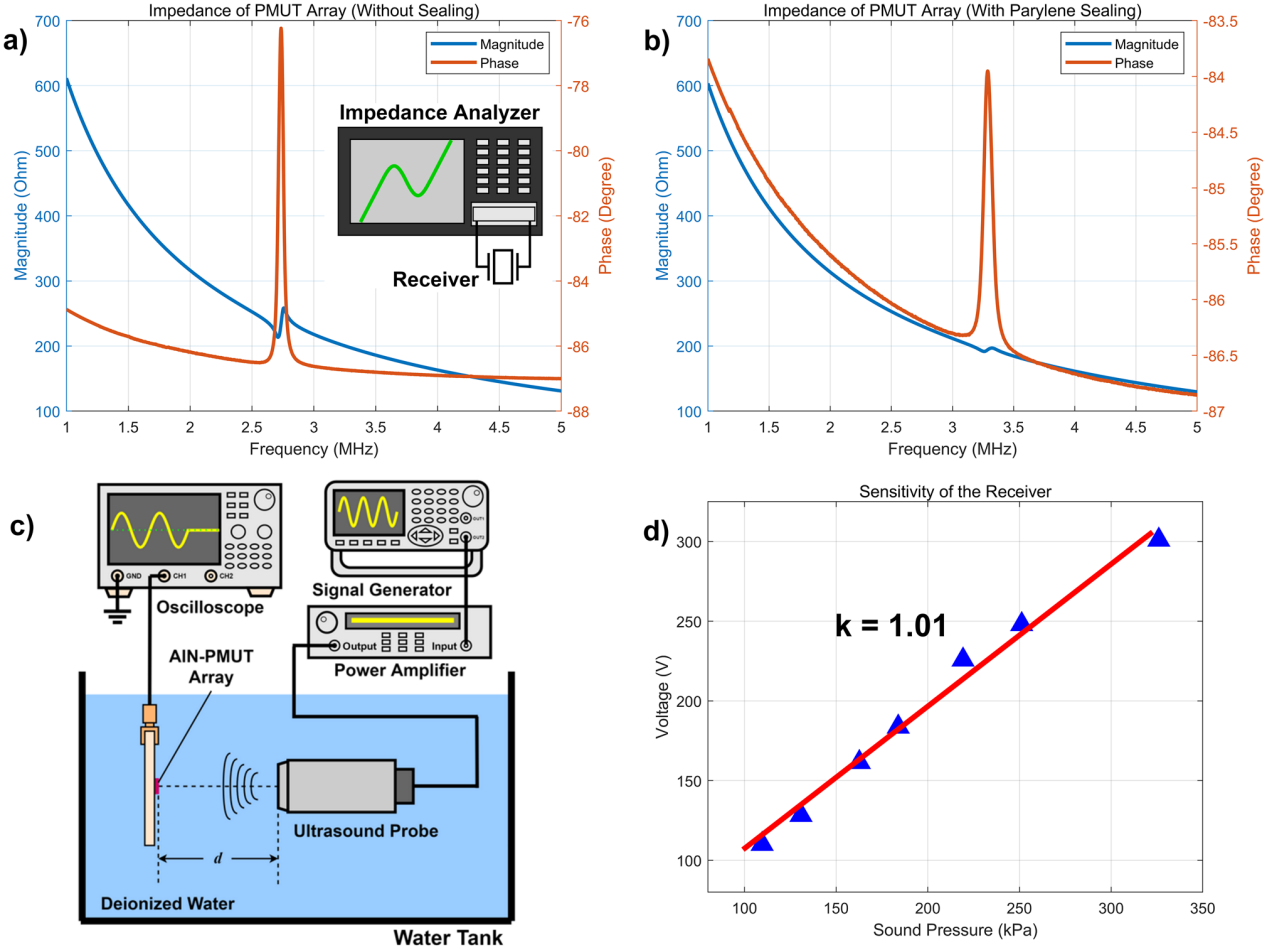
Test experiment of the PMUT array. (**a**) Curves of the PMUT array impedance without parylene sealing. (**b**) Curves of the PMUT array impedance with parylene sealing. (**c**) Experimental setup for evaluating AlN-PMUT array as wireless power receiver. (**d**) Measured sensitivity of the PMUT array. The sensitivity of the AlN-PMUT array was about 1 V/Mpa.

Electrical impedance curves of the receiver array were measured by an impedance analyzer before and after sealing. The measured results shown in Fig. 5a,b match well with the simulation value shown in Fig. 2c,d. By parallel connecting the PMUT elements, the electrical impedance was reduced to about 200 Ω.

Underwater receiving experiments were carried out characterize the receiving performance of the sealed PMUT array in a tank filled with deionized water, as shown in Fig. 5c. A function generator was connected to a power amplifier to generate a sinusoidal signal transmitted to the probe. The sound pressure generated by the commercial probe was calibrated with a needle hydrophone acquired from Precision Acoustics. We measured the receiving sensitivity of the receiver and performed a linear fitting to the data, as shown in Fig. 5d. The experiment results show that the receiving sensitivity of the PMUT is about 1 V/MPa.

The incident ultrasonic power intensity is controlled by the waveform of the function generator to 7 mW/mm^2^, which is below the FDA safety limit. The power intensity of the ultrasound wave can be calculated as:

2 $$\begin{array}{*{20}c} {I_{SPTA} = \frac{{p^{2} }}{2\rho c} \cdot C} \\ \end{array}$$

where *p* is the peak sound pressure of the ultrasound wave, *ρ* is the density of the propagation medium, *c* is the sound velocity in the medium and *C* is the duty cycle of the wave. According to the ultrasonic input power intensity (7 mW/mm^2^) and the effective area of the receiver (2.55 mm^2^), the input power is about 17.85 mW. According to the impedance and output voltage of the PMUT, the output power from PMUT can be calculated to be 42 μW, and the power transmission efficiency (PTE) is calculated to be 0.236%.

### Voltage multiplier design and optimization

**Figure 6:**
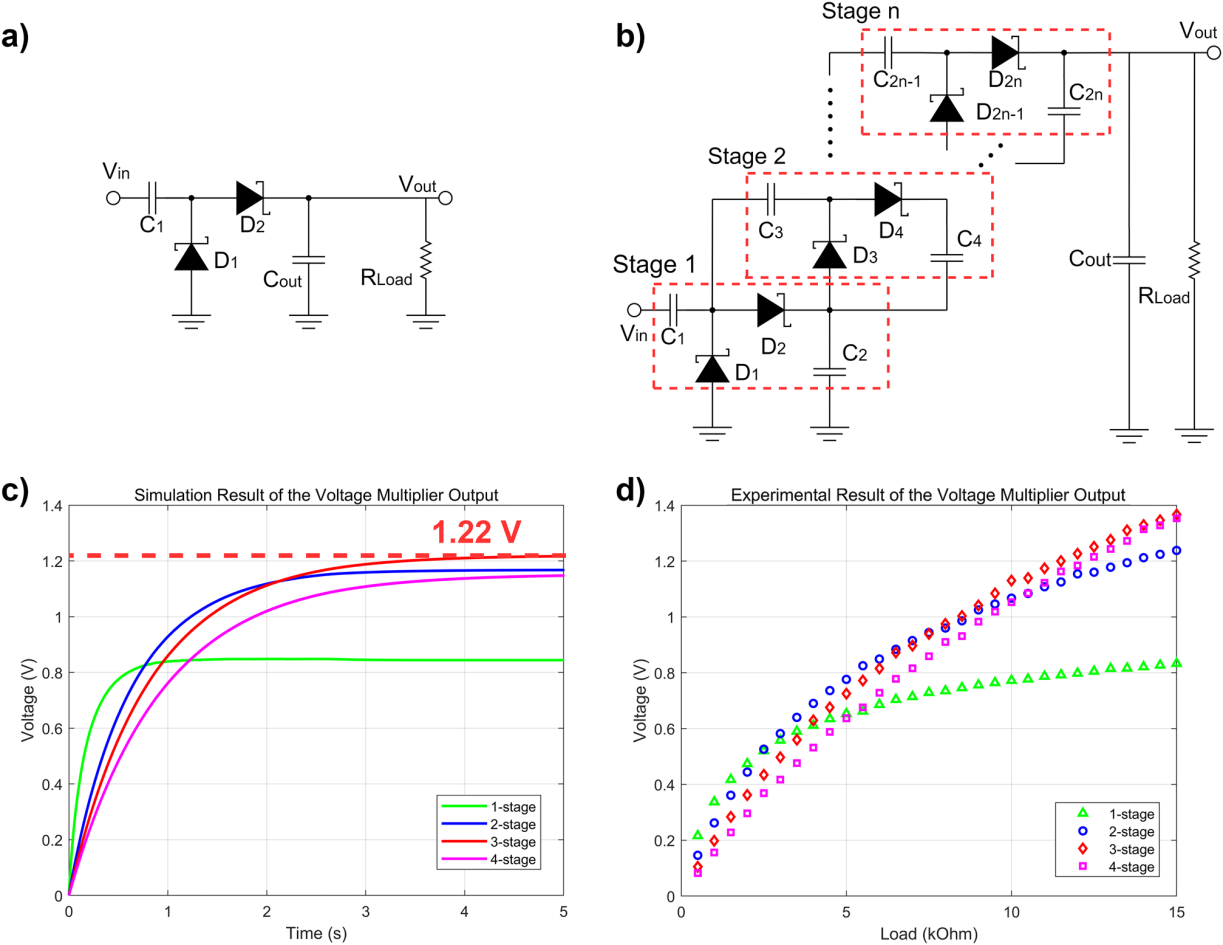
Characterization of the rectifier and boost circuit. (**a**) Schematic diagram of a single-stage voltage multiplier. (**b**) Schematic diagram of an n-stage Villard voltage multiplier. (**c**) SPICE simulation result for Villard voltage multipliers with a load resistance of 10 kΩ. The simulation result indicates that the 3-stage circuit achieves the maximum output power. (**d**) Experimental result for the Villard voltage multiplier output under different loads. The AlN-PMUT is directly connected to the voltage multiplier circuit.

The ultrasonic power received by the PMUT generates an AC signal. To convert AC into DC power, the voltage multiplier constitutes a crucial part of the WPS device. The output DC voltage level of a single-stage rectifier (Fig. 6a) is relatively low and insufficient for subsequent circuits or applications^26,27^. Compared to single-stage rectifiers, although voltage multipliers contain more components and thus attain a lower efficiency, n-stage voltage multipliers rectify the input signal and boost the voltage^28^.

Voltage multipliers exhibit many structures. In energy harvesting (EH) and WPT applications, the most commonly employed voltage multipliers include the Villard voltage multiplier (Cockcroft–Walton voltage multiplier) and Dickson voltage multiplier. The output voltage of these voltage multipliers depends on the number of stages and forward voltage of diodes. The power transmission efficiency of a given voltage multiplier is determined by the component performance, structure, input power level, load, source, and number of stages. A voltage multiplier could be implemented involving Schottky or CMOS diodes with transistors, and each stage consists of two capacitors and two diodes. We assessed and simulated the performance of different commercial Schottky diodes. The chosen diode was the 1SS372 diode purchased from Toshiba, which exhibits a very low forward voltage (0.18 V @ 1 mA), high switching speed and lead-free SOT-323 package and is suitable for low-power miniaturized applications. According to the simulation results, the performance levels of Villard and Dickson voltage multipliers are similar. A schematic diagram of an n-stage Villard voltage multiplier is shown in Fig. 6b.

To optimize the design, we simulated the performance of voltage multipliers with different structures and number of stages in SPICE software. With increasing number of stages, the input impedance of the multiplier decreased. We built a SPICE simulation model of the PMUT based on the impedance analysis results and simulated it together with the voltage multipliers. The input impedance of the power management circuit is about 10 kΩ, and power management circuit is directly connected to the output of the voltage multipliers. Therefore, 10 kΩ was chosen as the load of voltage multipliers. The simulation result is shown in Fig. 6c, revealing that the 3-stage voltage multiplier achieves the highest output of 1.22 V. Due to the internal resistance and capacitance of the PMUT array, voltage multipliers involving more than 3 stages could not produce higher output voltage levels.

To verify the simulation result, we built and evaluated different voltage multiplier circuits with the PMUT. To obtain more energy in the storage capacitor, we adopted a 100-μF capacitor as C_out_. The PMUT output was directly terminated with the input of the voltage multiplier. The experiment was also carried out in deionized water in a similar manner to the previous experiment. We assessed 1 to 4-stage voltage multipliers with different loads. The test result is shown in Fig. 6d. The output of the 3-stage voltage multiplier reached approximately 1.13 V. The margin of error between the simulation result and measured value was 11%. The experimental results indicate that the output voltage of the 3-stage voltage multiplier at 10 kΩ is higher than that of other circuits (0.77 V, 1.05 V and 1.07 V). Therefore, 3-stage voltage multiplier was adopted for the final design.

### Impedance matching network

**Figure 7:**
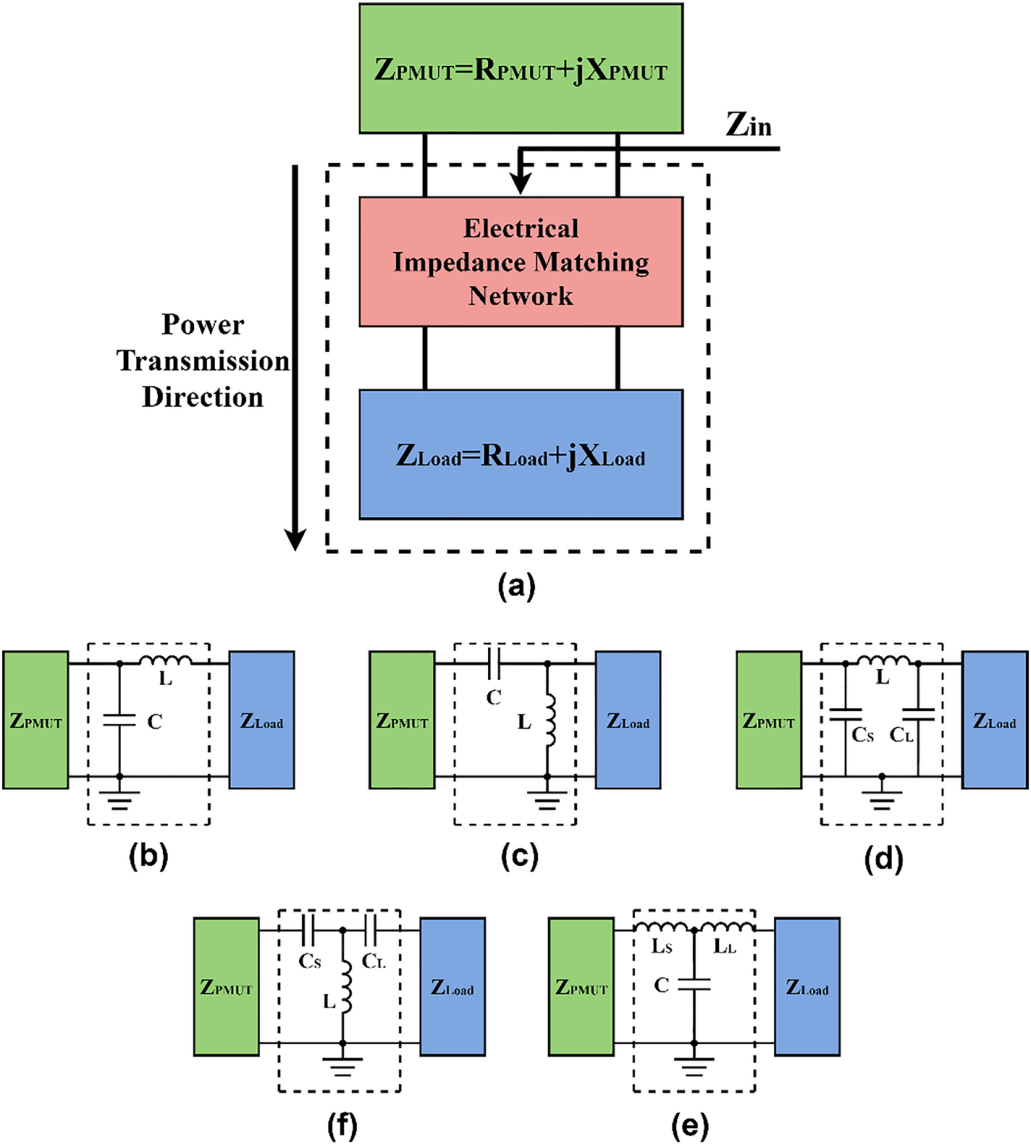
Impedance matching networks. (**a**) Schematic diagram of the impedance matching network. When the circuit is perfectly matched, Z_in_ is conjugate-matched with Z_PMUT_. (**b**) Low-pass L-type EIMN. (**c**) High-pass L-type EIMN. (**d**) Low-pass Pi-type EIMN. (**e**) Low-pass T-type EIMN. (**f**) High-pass T-type EIMN.

In transducer applications, the impedance matching method is usually applied to improve the signal-to-noise ratio (SNR), power level of the desired signal and bandwidth of the transducer^29,30^. In our application, we designed and evaluated several electrical impedance matching networks (EIMNs) to improve the power transmission efficiency between the receiver and subsequent circuit. The proposed electrical impedance matching networks were based on filter structures with passive components, including capacitors and inductors^31–33^. Maximum power transfer from the source to the load can be achieved if the load impedance is the complex conjugate of the source impedance^34^. As shown in Fig. 7a, the equations below must be satisfied:

3 $$\it {R_{PMUT} = Re( {{\text{Z}}_{{{\text{in}}}} } )}$$

4 $${X_{PMUT} = Im( {Z_{in} } )}$$

To design the above electrical impedance matching networks, the impedance values of the PMUT and subsequent circuit must be known. The measured impedance of the PMUT at excitation frequency is 24.1–j312.6 Ω, and the impedance of the circuit is 19.7–j979.7 Ω. The parameters of each EIMNs were automatically calculated by simulation software. We designed L-type and 3-element EIMNs in simulation software and assessed these circuits in the subsequent experiment. The designed L-type electrical impedance matching network included low and high-pass filter structures, as shown in Fig. 7b and c, respectively. In the low-pass filter structure, the calculated values of the inductor and the capacitor are 100.5 μH and 26.7 pF, respectively. In the high-pass matching network, the calculated values of the inductor and capacitor are 41 μH and 103.4 pF, respectively. Three-element EIMNs generally include Pi-type matching networks and T-type matching networks. As shown in Fig. 7d, the designed Pi-type matching network entailed a low-pass filter containing two shunt capacitors and one series inductor. The calculated values of C_S_ and C_L_ are 548.6 pF and 151.1 pF, respectively, and the value of the series inductor is 351.6 μH. The designed T-type matching network included low- and high-pass filter structures, as shown in Fig. 7e and f, respectively. The low-pass T-type matching network contained two series inductors and one shunt capacitor. The calculated values of LS and LL are 30 μH and 82.7 μH, respectively, and the value of the series capacitor is 2.3 nF. The high-pass T-type matching network included two series capacitors and one shunt inductor. The calculated values of C_S_ and C_L_ are 320.2 pF and 313.4 pF, respectively, and the value of the shunt inductor is 2.8 μH.

We measured the output DC voltage level of the voltage multiplier with different EIMNs. The output DC voltage without an EIMN reached 1.1 V. The L-type high-pass EIMN and T-type high-pass EIMN have the best performance, and increased the output voltage to approximately 1.4 V and 1.2 V, respectively. The error in the component value ranged from approximately 10–20%. Moreover, the direct current resistance (DCR) of the inductors could also affect the EIMN performance. The losses of capacitors are negligible, but the DCR of inductors usually reaches a few ohms. Because the DCR value is close to R_PMUT_ and R_Load_, the EIMN performance is degraded. Therefore, the L-type high-pass EIMN and T-type high-pass EIMN attained a better performance because these EIMNs include shunt inductors, minimizing the negative effects of the DCR. The output power of the voltage multiplier without EIMN reached 11.6 μW. The L-type high-pass EIMN and T-type high-pass EIMN increased the output power by approximately 60% and 19%, respectively, and the time-averaged output below the FDA safety limit reached 18.8 μW and 13.84 μW, respectively. The output power intensity is about 7.36 μW/mm^2^.

## Discussion

**Figure 8:**
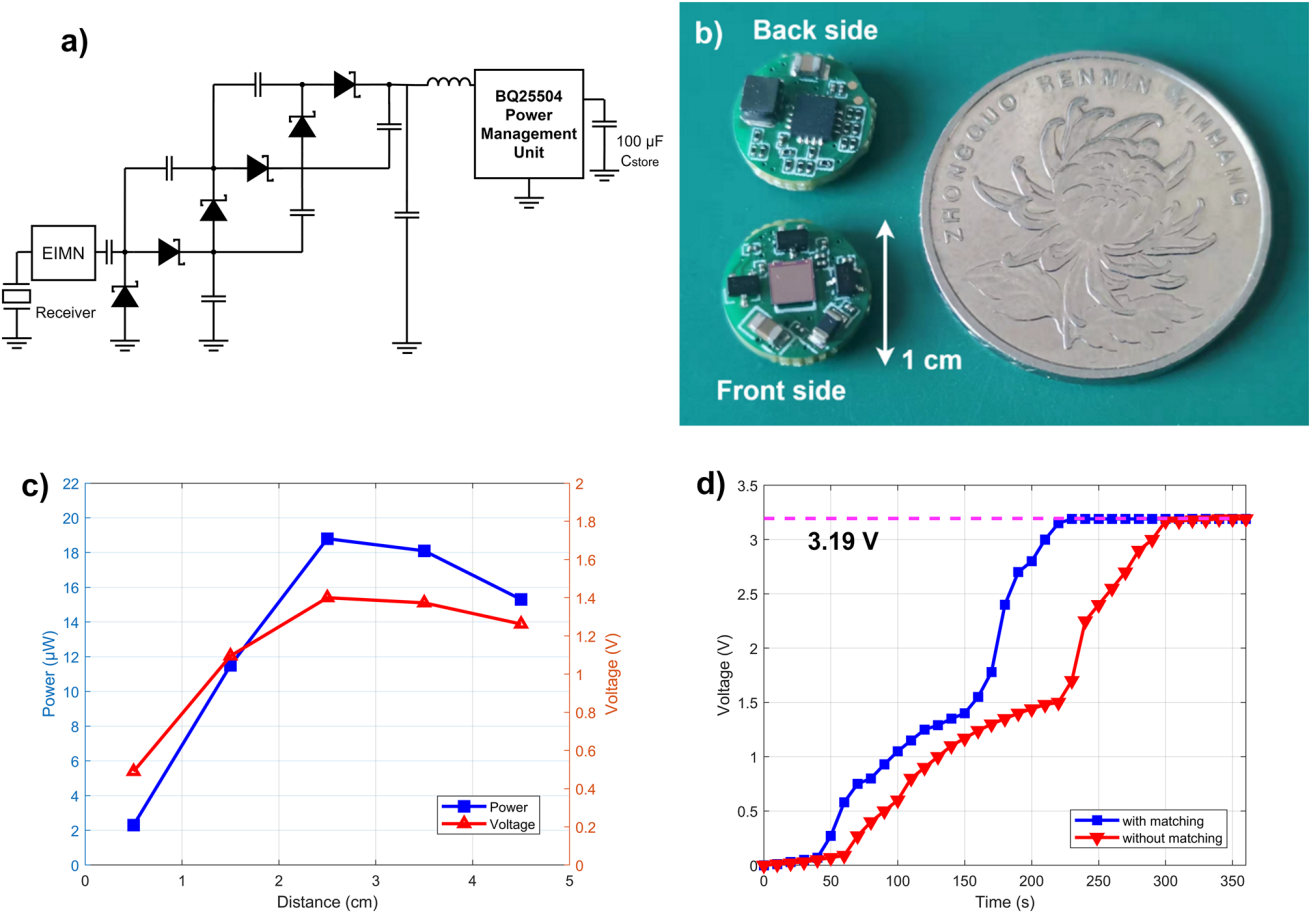
Proposed WPS device. (**a**) Schematic diagram of the proposed WPS device. (**b**) Photo of the proposed WPS device with a diameter of 1 cm next to a coin. (**c**) Received voltage and power of the voltage multiplier at different distance. (**d**) Charging curve of the 100-μF MLCC. It takes less than 4 min to charge a 100 μF MLCC to 3.19 V with impedance matching, in contrast to 5 min without impedance matching.

The proposed WPS device was implemented in a circular FR-4 PCB with a diameter of 1 cm fabricated through a lead-free process. The final circuit design discussed above is shown in Fig. 8a. The device includes the AlN-PMUT array, electrical impedance matching networks, voltage multiplier circuit, PMU and 100-μF MLCC storage capacitor on the PCB, as shown in Fig. 8b. Received voltage and power of the voltage multiplier output at different distance are shown in Fig. 8c. As shown in Fig. 8d, it takes about 4 min to charge a 100 μF MLCC to 3.19 V with impedance matching, faster than that without impedance matching.

The experimental PTE of the AlN-PMUT array in water is about 0.236% at a characterization depth of 25 mm, as shown in section “Voltage multiplier design and optimization”. The maximum power transmission distance and PET in tissue have also been estimated. The formula used for estimation is

5 $${p( x ) = p_{0} e^{ - \alpha ( f )x} }$$

where the attenuation factor α(*f*) is a function of operating frequency, *x* is the distance along the acoustic axis, *p*_0_ is the measured sound pressure of the probe, and *p* is the estimated sound pressure^4^. In blood and tissue, the attenuation coefficient (0.3 dB cm^−1^ MHz^−1^) of sound waves is generally higher than that in water. It is calculated that the maximum power transmission distance in blood or tissue is about 6 cm with the maximum PTE of about 0.13%.

For comparison purposes, related works in recent years on miniaturized acoustic WPT devices are summarized in Table 1. Table 1 only shows the related works of MEMS transducers and sub-millimeter PZT ceramics, larger WPT devices are not shown in the table. As the volume of wireless power receiver increases, its efficiency generally increases. The output power intensity, power delivered to load and PTE of our device reach 7.36 μW/mm^2^, 18.8 μW and 0.236%, respectively. They are already better than many PZT-based acoustic WPT devices^35–37^, although PTE and power delivered to load of our device are not the best^8,11^ due to inherent lower piezoelectric coefficient of AlN. Compared with other piezoelectric materials, ceramic PZT materials have higher piezoelectric constant and effective coupling coefficients, and therefore they generally have higher PTE. In addition, ceramic PZT are bulk material, and thus its higher quality factor at mechanical resonance would also improve the PTE value at the cost of lower bandwidth. The output power of our device reaches higher than 10 μW and the fully charged power supply voltage of our device reaches above 3 V. From practical use perspective, they are sufficient to power low-power IBDs for many applications, such as timers for biosensors (< 660 pW), neural electrical stimulations (> 1 μW), high data-rate intrabody communication or MEMS switches for implantable medical devices^37–42^. Furthermore, the AlN-based transducer in this work is lead-free, CMOS compatible and thinner in size, in contrast to state-of-the-art PZT-based transducers. The AlN PMUT-based WPS in this work will be used in our future research to neural electrical stimulation and passive communication for wireless recording of neural systems.

**Table 1:** Acoustic wireless power transfer devices.

	2014^35^	2016^36^	2016^37^	2017^8^	2018^11^	2019^38^	This work
Piezoelectric material	Thick PZT	Ceramic PZT	Thin PZT	Ceramic PZT	Ceramic PZT	(K,Na)NbO_3_ composite	Thin AlN
Lead-free transducer	No	No	No	No	No	Yes	Yes
CMOS compatible transducer	No	No	No	No	No	No	Yes
Experiment medium	Tissue	Water	Water	Oil	Oil	Tissue	Water
Receive area (mm^2^)	~ 10	~ 0.02	2.06	~ 3.8	0.36	1	2.55
Transducer thickness (μm)	500	127	8.2	950	600	600	7.45
Power intensity (μW/mm^2^)	4.9	N/A	0.041	N/A	N/A	4.5	7.36
Characterization depth (mm)	22	30	23	30	60	N/A	25
R_Load_ (kΩ)	0.5–4	10–100	N/A	2.5	0.4–10	1	1–15
Frequency (MHz)	0.04	5	0.17–0.82	1.1	1	6.06	3
PTE (%)	0.096	0.002	N/A	0.65	1.93	N/A	0.236
Impedance matching	No	No	No	No	Yes	No	Yes
Power delivered to load (μW)	49	0.51	0.0843	N/A	3000	4.5	18.8

Our future work will focus on optimization of the PMUT array and electrical impedance matching networks to achieve a higher output power level and power transmission efficiency, respectively. Furthermore, experiments in tissue will be performed with packaged devices for practical demonstration purposes. When implanted in animal body in future research, the WPS can be entirely packaged using biocompatible materials, e.g. parylene. Meanwhile, the PCB can be replaced by a biocompatible substrate, e.g. polyimide. Finally, due to the CMOS compatibility of the PMUT array, the circuits could be implemented in ASIC format and integrated with an AlN-PMUT array as a single chip in the future, whose size could be reduced to millimeters and even smaller. Even though the PMUT-CMOS monolithic chip has not yet been realized, the proposed solution in this work paves the way for ultraminiaturized, biocompatible and CMOS compatible wireless power supplies.

## Conclusion

This work introduced an ultrasound-induced WPS including WPT, power management and energy storage functions. The sensitivity of the AlN-PMUT array was about 1 V/Mpa and the PTE was about 0.236%. Electrical impedance matching networks were included to improve the power transmission efficiency. The output power intensity with rectification and a boost circuit reached 7.36 μW/mm^2^, and the charged voltage on the 100-μF capacitor could reach 3.19 V, which is sufficient for many implantable low-power sensors and ICs. The WPS was implemented in a PCB with a diameter of 1 cm. The proposed solution has the potential to be fully biocompatible and CMOS compatible when the AlN-PMUT array and CMOS circuit are integrated on a single chip in the future.

## Methods

### Characterizations and measurements

For the impedance measurement of the receiver, a E4990A impedance analyzer (Keysight, USA) was used. A DG4000 signal generator (RIGOL, China) was used to generate sinusoidal signals to the transmitter. For the sound pressure measurement, 2.0 mm needle hydrophone NH2000 (Precision Acoustics, UK) was used. For voltage measurement, the output was connected to a RTB2002 oscilloscope (Rohde & Schwarz, Germany).

## Data Availability

The data that support the findings of this study are available from the corresponding author upon reasonable request.
